# Supplementary material for: An enhanced multilevel secure data dissemination approximate solution for future networks
Source: PLoS One. 2024 Feb 8;19(2):e0296433. doi: 10.1371/journal.pone.0296433 (PMC10852309; doi:10.1371/journal.pone.0296433)
Supplement: Attachment — Submitted filename: SecureDissemination (PONE-D-23-10551) Reviews Report.pdf [file pone.0296433.s001.pdf]

SecureDissemination (PONE-D-23-10551) Reviews Report +1

OBSERVED AIMS OF THE STUDY

The authors seek to employ scheduling algorithms, with security category constraints, for data dissemination security.

TOPICAL COMMENTS WITH SUGGESTIONS

Title: *Routine-Based Algorithms for a Secure Data Dissemination Based on Security-Category Constraints*.

1. Title is NOT clearly stated (somehow clumsy).
2. SUGGESTION: The concepts upon which the security principle is based could be merged. Authors should NOTE that an unambiguous Title may attract better readership, for higher citations.

HINTS: modify the Title: *in accordance with the study; prominently highlighting the GAP identified in the reviewed existing similar and/or related works*.

Abstract:

The Abstract can NOT stand alone; as an accurate summary of the research; because:
1. NO specific weaknesses of the existing related works are mentioned.
2. The "different datasets" are NOT stated.
3. The algorithms used are NOT specified.
4. Which algorithms are the "other proposed algorithms"?
5. Which algorithm is "the best algorithm in the literature"?
6. Are the objects of comparisons outcomes/outputs of similar basic experimental conditions/environments?
7. The concluding statement does NOT justify the potential preferences of the proposed solution over the motivating limitations of the existing solutions.

HINTS: *restructure* the Abstract into: *why we must do what, what was actually done, how it was done, what result was obtained,* and *why the result was better*; and present in line with the Journal format.

Keywords:

1. In this paper, NO Keyword is stated.
2. It is academic to present a list of Keywords immediately after the Abstract.

HINTS: ensure the keywords reflect – *the implicit and explicit intents of the Title of the article*.

Introduction:

1. Generally, the Introduction is quite elaborate. However, the organization is NOT logical enough.
   For instance, the statement of the problem is expected to be the opening paragraph, because that is the rationale, which necessitated the proposed methods.
2. WITHOUT citation, statements like "… ten billion devices are connected today" are considered BOGUS.
3. Industry 5.0, OR 5.0 Industry, OR 5.0 Industry Revolution, OR 5IR; be accurately CONSISTENT.
4. NO specific security LEAKAGE is presented for NONE of the many data communication network issues raised in the single-paragraphed Introduction.
   a. NO specific Gap in literature is presented. What are their weaknesses/LIMITATIONS? What are scientifically responsible for these GAPs?
   b. NO specific existing solution (performance) is described as NOT satisfactory. Why are the proposed approaches considered capable of addressing the identified Gaps (in the existing related works)? (provide scientific JUSTIFICATIONS).
   c. Why NO algorithm, except scheduling, could represent the best solution for secure data dissemination is NOT justified.
5. Would the propose approach modifies the existing OSI model; as the authors are worry that "the OSI model's current design less effective in dealing with numerous attacks"? How?
6. Would the proposed methods functionally enhance Border Gateway Protocol; since the authors expressed concerns that "BGP is vulnerable to attacks"? How?

7. The rationale for proposing "… an intelligent private network solution that uses a security policy to provide a multilevel data dissemination control based on security category constraints …" is NOT justified.
   a. What is the "security policy"? Why is the policy considered "intelligent" and "private"?
   b. How many "multilevel controls"? Which particular "transmission controls" are multileveled; and how?
   c. What are the "security categories"? Which specific "category constraints" are applicable?
   d. How "the security category constraints" constitute an NP-hard problem is NOT clarified.
8. AMBIGUITIES: "While on one hand, the elaboration of the proposed solution relies on the scheduling algorithms presented in [6] [7] [8] [9] [10] [11] [12] [13], which are planned later to be utilized and adapted to the studied problem. On the other hand, applying the security category constraints is considered an NP-hard problem, which means that no algorithm will always efficiently produce the exact correct answer on all inputs. For that reason, the scheduling algorithm could represent the best choice to get the best solution …"
9. How are those existing algorithms [6-13] "… utilized and adapted to the studied problem …"?
10. If "…scheduling algorithm could represent the best choice to get the best solution in the current work …", why/how are "… the security category constraints …" functionally integrated into the scheduling framework?
11. Why is a "two-router network" proposed?
12. "The proposed two-router solution has many benefits: (i) introduces a security-by-design future network idea that relies on a dissemination-based packet classification; (ii) applies various algorithmic techniques such as dispatching rules, local insertion search, randomization method, and lifting procedure to enhance the security and performance of the transmitted packets in the computer network area; (iii) presents an idea that can be implemented as a private network application to assist individual privacy protection in critical environments and circumstances." "The disadvantages of the proposed method are as follows: (i) the times' complexity incurred from relying on a two-router solution that is a reduction from an NP-hard two-machine problem which demands using more techniques to solve it using algorithms with $O(n^3)$ heuristically. (ii) Developing an exact solution requires using a lower bound in a branch-and-bound algorithm."
    a. The motivation for the proposed methods is NOT clear; as benefit (ii) above is a set of different modules of the main approach, whose implementation are largely NOT reported; benefit (iii) is a mere future research projection; disadvantage (i) presents NO basis for the $O(n^3)$ time complexity; and disadvantage (ii) supposed to be the target approach, since it is possible to give the exact solution.
    b. NONE of the benefits NOR disadvantages is linked to any GAP in literature.

HINTS: begin the Introduction with – *a clear statement of the problem; how others have tried to solve the problem; how this paper improves on previous solutions. The motivation should be clearly highlighted, while showing the importance of the research. Strong and relevant key references are cited accordingly. Background scientific anatomical information is provided. The last statement should describe specifically what was done*.

Literature Review (Related Works):

1. The Literature reviews are generally vaguely presented; NOT concise; and, NOT coherent.
2. N.B.: Reviews are expected to be presented either literature-by-literature, or concept-by-concept, or principle-by-principle, or theory-by-theory, or approach-by-approach (each paragraph containing a single unit of discussion).
3. The methods for all the directly related works (that are relevant to the proposed methods) should be explicitly presented; especially, [14-16] supposed to be elaborately detailed; as the "works dealt with the studied problem" (according to the authors).
4. Review appraisals (which should precisely elucidate the specific Gap that motivated the proposed approach) are NOT presented. Thus, the necessity-proof for the proposed solution is NOT scientifically justified.

HINTS: organise each review into – *motivations, objectives, methods, results, strengths (knowledge advanced), and weaknesses (limitations – GAPS) of the work*.

Methodology:

1. How the approach minimizes transmission time is clear; as claimed in "… minimize the total transmission time over the two considered routers …"; but how such speed could translate to security is NOT presented.
2. NONE of the datasets generated is presented; as being claimed in "… experiments were conducted with different datasets generated according to uniform and binomial distributions …"
3. NO procedure is presented to justify that "… transmitted data is classified into different confidentiality levels … using … a non-classic (distinctive characteristics of data categories) traffic classification approach …"

4. NONE of the statistical properties (for statistical classification) is scientifically justified as a security attribute; as being claimed in "… utilising the statistical properties of the network traffic flow … for security purposes such as filtering traffic and identifying and detecting malicious activity …"
5. NO specific feature NOR features extraction procedure is presented; as being claimed in "… effectiveness of the classifier in statistical classification depends on the features extracted from the flow …"
6. NO procedure is presented on how "… two packets belonging to the same confidentiality level can't be transmitted through the two routers simultaneously …"; in furtherance to the authors claims.
7. What parameters are the determinants for each security level, and the number of possible security levels?
8. What determines the size of each packet? Are there limits to the possible number of packets?
9. What is the security constraint that prevents simultaneous transmission of data?
10. If "$P_{cs} = P_{cs1} \cup P_{cs2}$"; then, "$n_{pcs} = |P_{cs1}| \cup |P_{cs2}|$" is NOT accurate (NOT a correct math formulation).
11. Must the "security level" be always imposed and linked by the administrator? What are the standard guiding principles that the administrator must follow?
12. Upon which principle does the "any heuristic $h()$" operate?
13. What are the parameters that stipulate the "probability β"?
14. Until Example 1, there is NO mention of "the decreasing time algorithm": INCOHERENCY.
15. NONE of the seven proposed algorithms could appropriately characterise data transmission security; as DT (upon which all the remaining six algorithms are based) merely considers how long a process takes to complete: schedules from the longest process completion time to the shortest, engages the best possible number of processors, and maintains the order of the priority list, while possible idle times are minimized.
16. How the $ER2$ generation of sequences that are $DT - based$ translate to data protection of is NOT clear.
17. Algorithm 1 should be revised to reflect the independence of I_i on $CT_1 > CT_2$.
18. All examples should be within the context of proposed methods; since, according to the authors, "… experiments were conducted with different datasets generated …".
    a. Procedures should be described using datasets that are relevant to each experiment (fictitious datasets would NOT work in this study).
    b. All the examples, using fictive data, are evidences that NO experiment was carried out.

Authors should ensure that: *Methods answer ANY question around the aims and objectives of the study. Some of such questions are raised in the Introduction Section above. Methods should NOT be literature about concepts, but ACTIONS using applicable concepts; Except for references to the basis of the resulting experimental setups.*

Results and Discussions:

1. Results that are outcomes of fictive data would definitely be fictitious.
2. Thus, the results being claimed by the authors are NOT scientific, NOT academic, and NOT scholarly.

Authors should ensure Results discussions MUST answer the following/related questions: *What are the Findings? Which Finding justifies the expected outcomes? With what metrics were the quantity/quality measured? In the chosen subject DOMAIN, did the results meet the scope defined? Are there intermediate Results? Which background concepts are instrumental to the good outcome? Against which known work were the accuracy correlated? Is this outcome better? Does that outcome justify the motivating GAB? Whether the outcome is better or not, what future researches are suggested? (OPTIONAL).*

Discussions should be Relevant to – *the Results and Findings in the context of the Topic's objectives vis-à-vis related works; with answers to the research questions.*

Conclusions:

1. Emphasis on the Gaps in the previous related works, how the present study mitigates the Gaps, and the comparisons of their performances are NOT presented.
2. The Findings of the Results, which purportedly support the conclusions, are NOT outcomes of replicable experimental setups.
3. The suggested future researches are supposed to be the main target approaches of this study.

Authors should give expositions on the Findings in the conclusion; by: *reiterating the motivations and the identified gabs in a bid to declare the solutions being provisioned, while showcasing the additional knowledge contributions to the domain; giving justifications to whatsoever limitations identified, with suggestions for future works.*

References/Citations:

1. References are adequate; largely relevant; and just 68% recent.
2. Citations are orderly, and appropriately formatted.

Authors should be mindful of: *the Journal requirements; and simply strictly adhere to whatever citation and/or reference styles stipulated for submission and publishing processes*.

SPECIFIC COMMENTS AND SUGGESTED MODIFICATIONS

1. ***Readership***: is NOT good enough, as there are:
   a. Language (grammar) concerns that needs attentions;
   b. Indiscriminate use of "etc";
   c. Poor paragraphing (as in the Introduction and Related works sections);
   d. Sections are being referred to by non-existing numbers, as sections are NOT numbered;
   e. Some mnemonics are NOT defined at first instance (such as "PA", "NP-hard", "MDETA").

3. ***Manuscript Format***: Please, adhere strictly to the Journal formats (e.g., title, authors/affiliations, abstract, fonts/font-sizes, alignments, paragraphing, keywords, section-heads, subheads, sub-subheads, citations, tables, lists/bulleting, abbreviations, figures, graphics, equations, reference listings).

RECOMMENDATIONS

The research idea is good. However, ideas could NOT be academically substantiated; motivations are largely NOT based on articulated Gaps in literature; datasets are fictive; and NO scientific experiment could be linked with the authors claims in the Introduction and the professed Results. Hence, **the authors are encouraged to pay diligent attentions to the above comments, to ensure a CONSISTENT, COHERENT, and LOGICALLY organized ideas, which are scientifically proven and scholarly presented**.
