# Supplementary material for: An enhanced multilevel secure data dissemination approximate solution for future networks
Source: PLoS One. 2024 Feb 8;19(2):e0296433. doi: 10.1371/journal.pone.0296433 (PMC10852309; doi:10.1371/journal.pone.0296433)
Supplement: Attachment — Submitted filename: PONE-D-23-10551_R2.docx [file pone.0296433.s003.docx]

The manuscript has been improved significantly to meet the publication standards. Meanwhile, the referencing of the supporting literature was done inaccurately. For instance, it is wrong to ascribe [16] to the first paper cited in the introduction. Therefore, authors are advised to adopt the reference style specified on the journal’s portal.
