# Supplementary material for: An enhanced multilevel secure data dissemination approximate solution for future networks
Source: PLoS One. 2024 Feb 8;19(2):e0296433. doi: 10.1371/journal.pone.0296433 (PMC10852309; doi:10.1371/journal.pone.0296433)
Supplement: Attachment — Submitted filename: Reviwer Responses R2.docx [file pone.0296433.s004.docx]

Querry 1:

The manuscript has been improved significantly to meet the publication standards. Meanwhile, the referencing of the supporting literature was done inaccurately. For instance, it is wrong to ascribe [16] to the first paper cited in the introduction. Therefore, authors are advised to adopt the reference style specified on the journal’s portal.

**Answer:**

Done

Querry 2:

Please ensure that you refer to Table 7 in your text as, if accepted, production will need this reference to link the reader to the Table.

**Answer:**

Done
